# Transient Postoperative Hypoxemia Associated With Cannabis Use Following General Anesthesia

**DOI:** 10.7759/cureus.110926

**Published:** 2026-06-15

**Authors:** Shairko Missouri, Osman Elsiddig, Jessica Perkins

**Affiliations:** 1 Anesthesiology and Perioperative Medicine, Boston Medical Center / Chobanian and Avedisian School of Medicine, Boston, USA

**Keywords:** cannabis use, complications, general anesthesia, perioperative hypoxia, vaping

## Abstract

Cannabis use has increased worldwide, particularly with the rising availability of vaping products. While the cardiovascular and neurocognitive effects of cannabis are increasingly recognized in the perioperative setting, its pulmonary consequences remain less well defined. Cannabis inhalation has been associated with airway irritation, bronchial hyperreactivity, alveolar inflammation, and impaired gas exchange, which may predispose patients to perioperative respiratory complications. General anesthesia may exacerbate these subclinical abnormalities through reductions in functional residual capacity, atelectasis, and altered pulmonary perfusion. These mechanisms may unmask underlying disturbances in gas exchange. We report a case of transient postoperative hypoxemia following general anesthesia in a young patient with heavy cannabis vaping exposure and no known cardiopulmonary disease. The patient developed hypoxemia with minimal radiographic findings and preserved ventilation, requiring supplemental oxygen postoperatively. His condition resolved spontaneously within 24 hours. This case suggests that clinicians should consider cannabis-related pulmonary effects in the differential diagnosis of postoperative hypoxemia.

## Introduction

Cannabis use is increasingly prevalent, with 64.2 million Americans reporting past-year use in 2024, and smoking remains the most common route of administration. Emerging evidence suggests that cannabis use disorder is associated with a modest increase in perioperative adverse outcomes, including respiratory complications. Inhaled cannabis exposure has been linked to airway inflammation, bronchial hyperreactivity, alveolar injury, and e-cigarette or vaping-associated lung injury (EVALI), resulting in pulmonary changes that may increase susceptibility to perioperative hypoxemia and other respiratory complications [1-3].

In the anesthetic literature, perioperative airway complications related to cannabis use, including bronchospasm, wheezing, and delayed emergence, have been described [4,5]. Chronic cannabis use has also been associated with structural and functional pulmonary changes, including airway inflammation and impaired gas exchange [6]. These effects may remain subclinical and unrecognized, particularly in otherwise healthy individuals, yet may become clinically significant in the perioperative setting.

More recent evidence from EVALI has further highlighted the potential for cannabis vaping to cause significant pulmonary dysfunction, including hypoxemia that may occur despite minimal or normal radiographic findings [3,7-9]. Importantly, these pathophysiologic changes may result in impaired gas exchange that is not readily apparent on imaging but can manifest clinically under physiologic stress.

General anesthesia may unmask these underlying abnormalities through reductions in functional residual capacity, atelectasis formation, and alterations in pulmonary perfusion, thereby exacerbating ventilation-perfusion mismatch. As a result, patients with recent or heavy cannabis vaping exposure may develop postoperative hypoxemia despite preserved ventilation and an otherwise uncomplicated intraoperative course. In many cases, this phenomenon appears to be transient and self-limited, with resolution occurring within 24-72 hours as pulmonary function recovers, consistent with the reversible nature of anesthesia-induced atelectasis and associated gas exchange impairment [10,11].

Given the increasing prevalence of cannabis use and vaping, these observations underscore the importance of careful preoperative screening for cannabis exposure and consideration of its potential pulmonary effects. Increased awareness among anesthesiologists and perioperative clinicians may facilitate early recognition, appropriate monitoring, and risk mitigation strategies in patients at risk for cannabis-associated respiratory complications.

We present a case of transient postoperative hypoxemia following general anesthesia in a young patient with heavy cannabis vaping exposure and no known underlying pulmonary disease.

## Case presentation

A 33-year-old male with a history of obesity (body mass index 35 kg/m²) and no known cardiopulmonary disease underwent robotic excision of a small oropharyngeal mass. The patient reported vaping cannabis two to four times daily and smoking cannabis in the morning of surgery. It is unknown how many years of exposure he had to cannabis.

Based on the patient’s body mass index (BMI) and airway exam, a preoperative obstructive sleep apnea (OSA) assessment was done and resulted in a STOP-Bang score of 5 (STOP: snoring, tiredness, observed apnea, and high blood pressure; Bang: BMI, age, neck circumference ≥40 cm, and male gender), with points scored for loud snoring, daytime tiredness, witnessed apneic episodes, BMI>35 kg/m², and male gender. The patient was referred for a sleep study at the hospital. He had six metabolic equivalents (METS) when questioned about his exercise tolerance, and he only experienced shortness of breath when walking long distances.

After the preoperative assessment was completed, the patient was brought to the operating room and general anesthesia was induced with intravenous lidocaine 100 mg, fentanyl 100 mcg, propofol 200 mg, and rocuronium 70 mg. The patient was mask-ventilated using a nasal airway. Nasotracheal intubation was performed using videolaryngoscopy with a grade 1 view. Anesthesia was maintained with propofol and remifentanil infusions that were titrated to a goal processed electroencephalogram reading of 30-50. The patient received 0.5 mg/kg of ketamine for a total of 60 mg, and he received 20 mcg of dexmedetomidine in increments of 10 mcg for analgesia and sedation. He did not receive any long-acting sedatives or opioids. The intraoperative course was uneventful, and vital signs were stable throughout the procedure. His respiratory parameters throughout the case were as follows, and he maintained an oxygen saturation of 96-98% throughout the case: FiO_2_ = 0.3, FGF = 2 L/min, VT = 460 ml, RR = 16/min, PEEP = 6 cmH2O, I:E = 1:2, ETCO_2_ = 37 mmHg, mean airway pressure = 10 cmH_2_O (*TV = tidal volume calculated based on a 6-8 cc/kg ideal body weight).

Neuromuscular blockade was reversed with sugammadex. A quantitative neuromuscular monitor was used, and the patient had four twitches without fade prior to extubation. All other extubation criteria were met. 

Emergence was complicated by severe agitation for which the patient received an additional 20 mcg of dexmedetomidine. Post-extubation, the oxygen saturation decreased to 80%. A nasal airway was placed, and the patient remained spontaneously ventilating with tidal volumes of approximately 1200 cc on 100% FiO_2_. Lung sounds were clear and diminished. The patient’s oxygen saturation improved following recruitment maneuvers, and he was transitioned to 8 L/min oxygen via a simple face mask. His oxygen saturation remained greater than 95%, and he was transported to the recovery unit on this same oxygen concentration.

In the recovery unit, the patient required continuous oxygen supplementation to maintain oxygen saturation greater than 95%. He denied shortness of breath or difficulty breathing, and he remained ambulatory. The patient consistently achieved 1750 cc using the incentive spirometer, but he did not have any improvement in his oxygen saturation. 

**Figure 1:**
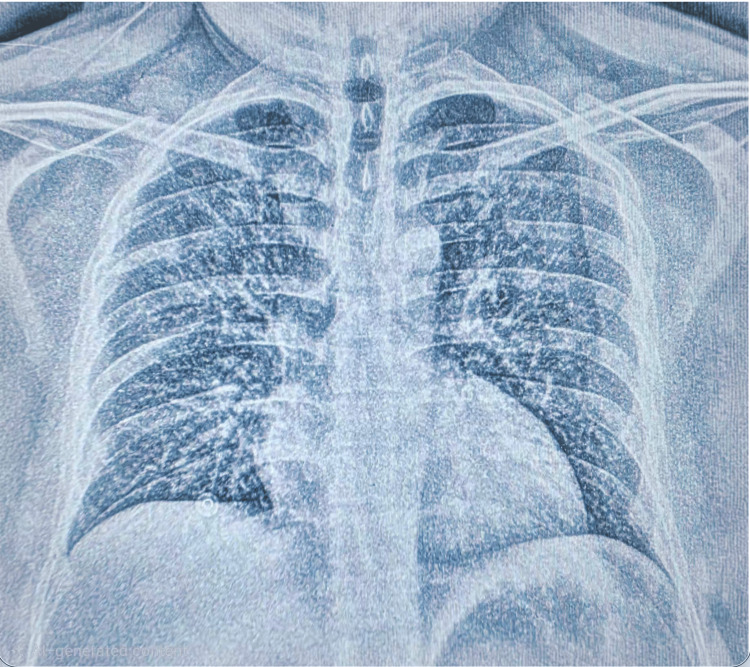
Anteroposterior chest X-ray: portable chest X-ray showing hypermarkings of vascular airways

Chest radiography showed no acute pathology but revealed increased pulmonary vascular markings (Figure 1).

Arterial blood gas analysis was as follows: pH 7.36, pCO_2_ 42.0, HCO_3_ 24.0, pO_2_ 57, O_2_ saturation 88, base excess -2, and Hgb 15.3. Intraoperatively, there was low concern for an aspiration event or negative pressure pulmonary edema. Internal medicine admitted the patient for observation. The patient continued to use the incentive spirometer and was started on an albuterol inhaler q4h PRN. His oxygen requirement resolved within 24 hours, and he was discharged without sequelae.

## Discussion

Pulmonary complications associated with inhaled cannabis use are increasingly recognized, although perioperative data remain limited. Chronic cannabis smoking has been shown to cause airway inflammation, increased bronchial reactivity, and impaired gas exchange [1,2,6], potentially predisposing patients to perioperative respiratory instability. These effects may be present even in young individuals without clinically apparent lung disease.

The pathophysiology of cannabis vaping-associated pulmonary dysfunction is multifactorial and shares features with EVALI. Inhalation of aerosolized cannabis products exposes the respiratory epithelium to ultrafine particles, lipid solvents, and thermal degradation products. Compounds such as vitamin E acetate, identified in bronchoalveolar lavage fluid in EVALI patients, have been implicated in the disruption of alveolar surfactant function and impairment of gas exchange [7]. In addition, vaping aerosols can induce oxidative stress, epithelial injury, and activation of alveolar macrophages, resulting in cytokine-mediated inflammation and increased alveolar-capillary permeability [8,9]. These processes may lead to ventilation-perfusion (V/Q) mismatch and impaired oxygen diffusion without necessarily producing overt radiographic abnormalities in early or mild disease.

EVALI has been reported to present with hypoxemia that may be disproportionate to imaging findings. Layden JE et al. described a spectrum of radiographic presentations ranging from normal to subtle interstitial changes in early disease [3]. Subsequent studies suggest that functional impairment in gas exchange may precede structural abnormalities detectable on imaging [9]. This concept is particularly relevant in the perioperative setting, where subtle preexisting abnormalities may be unmasked.

General anesthesia may further exacerbate these subclinical pulmonary changes. Anesthetic-induced reductions in functional residual capacity, atelectasis formation, altered pulmonary perfusion, and suppression of hypoxic ventilatory drive can worsen underlying V/Q mismatch. In patients with cannabis-related airway inflammation or endothelial dysfunction, these effects may synergistically impair oxygenation, resulting in clinically significant postoperative hypoxemia despite preserved ventilation.

In the anesthetic literature, cannabis use has been associated with perioperative airway complications, including coughing, wheezing, bronchospasm, and delayed emergence [4,5]. Emerging clinical observations suggest that inhaled cannabis exposure may also contribute to transient postoperative hypoxemia, even in the absence of structural lung disease, likely through inflammatory or endothelial-mediated mechanisms [5,9,10]. Similar patterns of reversible hypoxemia have been described in patients with mild inhalational lung injury and early EVALI presentations, where oxygen requirements resolve within 24-72 hours as inflammation subsides.

In the present case, several features support this mechanism, including heavy daily cannabis vaping, cannabis use on the day of surgery, persistent hypoxemia despite preserved ventilation and ambulation, minimal radiographic findings, and rapid resolution within 24 hours. The temporal relationship between exposure, anesthesia, and recovery strongly suggests a reversible functional disturbance rather than structural lung injury.

Several alternative causes of postoperative hypoxemia were considered and deemed less likely based on the clinical presentation. Aspiration was unlikely given the absence of intraoperative events, lack of radiographic infiltrates, and rapid clinical resolution. Negative-pressure pulmonary edema was also considered; however, there was no evidence of airway obstruction, laryngospasm, or characteristic radiographic findings. Opioid-induced hypoventilation was unlikely given preserved mental status, adequate respiratory effort, and absence of hypercapnia on arterial blood gas analysis. Pulmonary embolism was considered but deemed unlikely due to the acute onset immediately following emergence, normal hemodynamics, absence of risk factors or symptoms such as chest pain, and rapid resolution without anticoagulation. Although obesity raised the possibility of undiagnosed obstructive sleep apnea, the persistence of hypoxemia despite full wakefulness and ambulation argues against this as the primary etiology.

Collectively, these findings support a transient and reversible impairment in pulmonary gas exchange, most consistent with cannabis-related inflammatory and endothelial dysfunction, likely mediated through V/Q mismatch and alveolar-capillary barrier disruption, unmasked by the physiologic effects of general anesthesia.

## Conclusions

Cannabis vaping may contribute to subclinical pulmonary inflammation and impaired gas exchange that can be unmasked in the perioperative setting. General anesthesia may exacerbate these underlying abnormalities through reductions in functional residual capacity, atelectasis, and alterations in pulmonary perfusion, resulting in transient postoperative hypoxemia even in patients without known lung disease.

The rapid resolution observed in this case supports a reversible functional mechanism rather than structural lung injury. Increased awareness of cannabis-related pulmonary effects is essential for perioperative clinicians. Careful preoperative screening for cannabis use, consideration of abstinence prior to surgery, and vigilant postoperative monitoring may help reduce respiratory complications and improve patient safety.

Further prospective and controlled studies are needed to better characterize the pulmonary pathophysiologic effects of cannabis, particularly vaping formulations, and to establish clear perioperative risk stratification frameworks. Identification of patients at increased risk for postoperative respiratory complications will be critical to guide preoperative optimization strategies and perioperative management. Improved understanding of cannabis-associated pulmonary dysfunction may ultimately help minimize perioperative morbidity and inform evidence-based recommendations for surgical patients.
